# A microscopic look at the Johari-Goldstein relaxation in a hydrogen-bonded glass-former

**DOI:** 10.1038/s41598-019-50824-7

**Published:** 2019-10-04

**Authors:** F. Caporaletti, S. Capaccioli, S. Valenti, M. Mikolasek, A. I. Chumakov, G. Monaco

**Affiliations:** 1 Dipartimento di Fisica, Università di Trento, I-38123 Povo, Trento Italy; 2 Dipartimento di Fisica, Università di Pisa, Largo Bruno Pontecorvo 3, I-56127 Pisa, Italy; 3 CNR-IPCF, Largo Bruno Pontecorvo 3, I-56127 Pisa, Italy; 4 Grup de Caracterització de Materials, Department of Physics, Universitat Politècnica de Catalunya, EEBE, Av. Eduard Maristany 10-14, E-08019 Barcelona, Spain; 5 ESRF-The European Synchrotron, CS40 220, 38043 Grenoble, Cedex 9 France; 6 National Research Center “Kurchatov Institute”, 123182 Moscow, Russia

**Keywords:** Chemical physics, Phase transitions and critical phenomena, Statistical physics

## Abstract

Understanding the glass transition requires getting the picture of the dynamical processes that intervene in it. Glass-forming liquids show a characteristic decoupling of relaxation processes when they are cooled down towards the glassy state. The faster (*β*_*JG*_) process is still under scrutiny, and its full explanation necessitates information at the microscopic scale. To this aim, nuclear *γ*-resonance time-domain interferometry (TDI) has been utilized to investigate 5-methyl-2-hexanol, a hydrogen-bonded liquid with a pronounced *β*_*JG*_ process as measured by dielectric spectroscopy. TDI probes in fact the center-of-mass, molecular dynamics at scattering-vectors corresponding to both inter- and intra-molecular distances. Our measurements demonstrate that, in the undercooled liquid phase, the *β*_*JG*_ relaxation can be visualized as a spatially-restricted rearrangement of molecules within the cage of their closest neighbours accompanied by larger excursions which reach out at least the inter-molecular scale and are related to cage-breaking events. In-cage rattling and cage-breaking processes therefore coexist in the *β*_*JG*_ relaxation.

## Introduction

A way of tackling the long-standing problem of the microscopic origin of the glass transition is to study the dynamics of undercooled liquids on lowering their temperature (*T*) toward the glassy state^1^. Two main dynamical processes are common to all glass-formers: the *α* and the *β*_*JG*_ relaxation^1–4^. The *α*, or structural, relaxation has a characteristic time strongly dependent on *T* and spans many orders of magnitude in time, being about 100 s at the glass-transition temperature, *T*_*g*_. The *β*_*JG*_, or Johari-Goldstein, relaxation is less *T*-dependent, and decouples from the structural one below a temperature where the relaxation time is usually in the *μ*s - ms range, and remains active also in the glassy state. It is nowadays established that the *β*_*JG*_ relaxation is strictly related to and acts as precursor of the *α* relaxation which has a slower dynamics due to a larger cooperativity^1,3,5^. It is also clear that the *β*_*JG*_ process involves motions of the molecular unit as a whole^4^ and should not be confused with intra-molecular processes such as the motion of pendant groups in polymers. However, there is still no definitive microscopic description available for the *β*_*JG*_ relaxation, despite it being at the focus of a large number of phenomenological and theoretical studies, as well as of experiments and simulations^3–21^.

From a materials science perspective, the *β*_*JG*_ relaxation is claimed to play a role in a number of relevant properties: for example, when the *β*_*JG*_ relaxation is pronounced, metallic glasses show a high tensile plasticity^22,23^, and amorphous pharmaceuticals easily crystallize, which implies a reduction of the solubility of the medicine^24^. Therefore, unveiling the microscopic mechanism underlying the *β*_*JG*_ process is not only a crucial step toward a complete theory of the glass transition but is also of significance for many technologies and practical applications.

Most of what we know of the *β*_*JG*_ relaxation derives from dielectric spectroscopy (DS) and nuclear magnetic resonance (NMR) studies. DS, which is mainly sensitive to molecular reorientations^25^, has provided characteristic timescales as well as activation energies and relaxation strengths, i.e. the fraction of molecules participating in the relaxation process. Large-angle molecular reorientations are generally thought to be dominant at high temperatures^26^, and to become more restricted as *T*_*g*_ is approached from above. 1D and 2D NMR studies on small, organic molecules and polymers^7^ show indeed that below *T*_*g*_ the *β*_*JG*_ relaxation consists of small-angle (<10°), hindered reorientations. Consistently, a restricted reorientational diffusion is observed across *T*_*g*_^7^. Not much is known, instead, on the center of mass (CM) dynamics at the microscopic scale, which is crucial in order e.g. to learn about the typical length-scale and degree of cooperativity of the *β*_*JG*_ process. Probing in fact the relatively slow dynamics of this process at the microscopic length scale using numerical simulations and in experiments is extremely challenging.

On the experimental side, only a few glass-formers have been investigated up to now with the aim of studying the CM dynamics within the *β*_*JG*_ relaxation at the microscopic scale. Neutron scattering experiments on the polymeric system polybutadiene (PB) report evidence for the *β*_*JG*_ relaxation only at the intra-molecular scale^6,27^, and have been interpreted in terms of a model which predicts a strong wave number (*q*) dependence for the *β*_*JG*_ relaxation strength. Nuclear *γ*-resonance time-domain interferometry (TDI) experiments on PB^16^ and on the molecular glass-former o-terphenyl (OTP)^11,17^ confirm the existence of the *β*_*JG*_ relaxation at the intra-molecular scale, and demonstrate for OTP that it corresponds to a restricted dynamics^11,17^. More recent incoherent elastic and inelastic neutron scattering experiments on propylene carbonate analysed using an heterogeneous dynamics model suggest that the *β*_*JG*_ relaxation is characterized by a mean square displacement of about 0.5 Å and should therefore be associated to metabasin transitions in a potential energy landscape description of the undercooled liquid^19^. It would be important to generalize this conclusion to more glass-formers and, possibly, using experimental schemes requiring less, or at least different, model hypotheses. It is also interesting to underline that this result is in agreement with simple estimates for the molecular excursions within the *β*_*JG*_ relaxation process based on the local validity of the Stokes-Einstein relation and on the typical values of molecular reorientations obtained by NMR^7^. Numerical simulations based on a specially designed model suggest that, in the glass state well below *T*_*g*_, the *β*_*JG*_ relaxation is associated to large reorientations of otherwise immobile molecules^20^. In the simpler case of metallic glasses, computer simulations of long-enough atomic trajectories indicate moreover that cooperative rearranging regions with string-like shape are at the origin of the *β*_*JG*_ process, and rather identify the inter-molecular distance as the distinctive length-scale of the process^18,21^. The connection between string-like rearranging regions and the *β*_*JG*_ relaxation in metallic glasses is also supported by the finding that alloys with constituting atoms characterized by similar negative enthalpy of mixing present pronounced *β*_*JG*_-relaxation peaks in mechanical loss spectra: this indeed favours the formation of molecule-like structures^13^. This picture is in agreement with the idea proposed in Ref.^5^ of a distribution of processes with increasing participation of molecules and longer length scale with increasing time, and is also consistent with the predictions of the random first order theory^10^.

In order to shed light on this topic, we report in this work the results of a time domain interferometry (TDI) experiment performed on 5-methyl-2-hexanol (5M2H, *T*_*g*_ = 154 K), a monohydroxy alcohol^28^ belonging to the class of H-bonded glass-formers. 5M2H is characterized by a genuine *β*_*JG*_ relaxation^29^, as suggested by the strong pressure dependence of its characteristic time and by the change in activation energy occurring at *T*_*g*_^30^, evidence of a close connection of this process to the *α* relaxation. 5M2H was chosen since investigations of the *β*_*JG*_ relaxation at the microscopic scale have not yet been reported for hydrogen-bonded glass-formers such as mono-hydroxyl alcohols. Furthermore 5M2H has the advantage, with respect to already studied systems such as OTP^11^ or polybutadiene^16^, of displaying a *β*_*JG*_ relaxation exceptionally separated in timescale from the structural one well above *T*_*g*_^29,30^, which makes it ideal for the TDI dynamic range.

The study reported here of the *q*-dependence of the *β*_*JG*_ relaxation time clearly suggests its sub-diffusive nature, similarly to the OTP case^11^. However, differently from what observed in OTP^11^ and PB^16^, the *β*_*JG*_ process in 5M2H also appears at the inter-molecular scale, while it is clearly the dominating relaxation channel only at the intra-molecular scale. Moreover, by comparing the *q*-dependent TDI data (sensitive here to center-of-mass dynamics) to DS ones (sensitive to rotational dynamics), we manage to extract a characteristic length of 0.3 Å associated to the *β*_*JG*_ process. Our data thus clarify that, from the center-of-mass dynamics perspective, the *β*_*JG*_ relaxation consists, in average, of a restricted molecular translational dynamics characterized by CM rearrangements within the cage formed by the neighbouring molecules over a length scale of the order of 10% of the intermolecular distance. The spatial distribution of these rearrangements, however, has tails extending to longer distances which reach out at least the inter-molecular scale, thus reconciling the apparent contradictions of previous studies.

## Results

### Time-domain interferometry beating patterns

In a typical TDI experiment two nuclear absorbers, characterized by different nuclear energy spectra, are placed upstream and downstream of the sample^31^. Synchrotron radiation photons impinging onto the first nuclear absorber can either coherently excite the nuclear resonance or be simply transmitted. These two paths are coherently coupled and, after the interaction with the sample, recombine at the second absorber, which has a slightly different excitation energy and acts as a phase-sensitive analyzer. From the interference of these scattering paths from the probe (upstream) and reference (downstream) absorbers, a time-domain pattern of quantum beats arises. Since the coherent superposition of the paths is modulated by the scattering from the sample placed in between the two absorbers, when an energy transfer occurs (i.e. via quasi-elastic scattering) a loss of phasing and a consequent damping of the beating pattern is observed.

**Figure 1:**
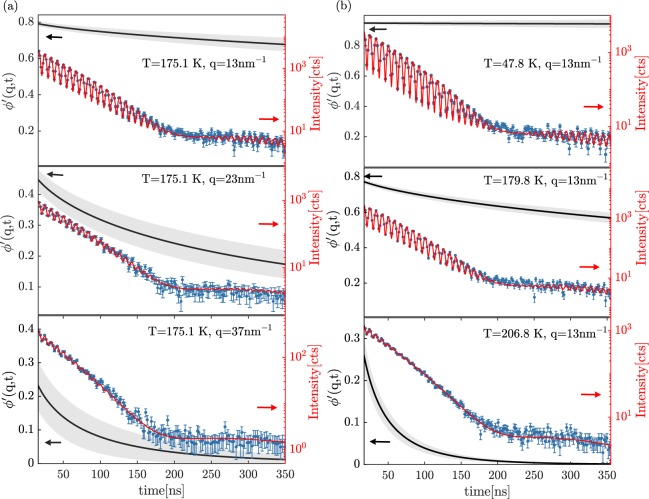
Time-domain interferometry patterns as a function of time (points with errorbars) at different exchanged wave-numbers *q* and at the same temperature *T* = 175.1 K in the undercooled liquid state (**a**) and at different temperatures at the same *q* = 13 nm^−1^ corresponding to the inter-molecular distance (**b**). The raw data have been averaged over a time range ±0.7 and ±0.9 ns, depending on the collected statistics, in order to improve the figure readability. The red-solid lines (right y-scale) are the model curves fitted to the data whereas the black solid lines (left y-scale) are the contrast functions, calculated from the fitting parameters along with the 68% confidence intervals (gray area).

Figure 1 shows some examples of the measured TDI patterns as a function of time at different *q*’s for a single *T* (a) and at different *T*’s for a single *q* (b). These beating patterns are modulated by the sample dynamics, and can be modeled in detail to extract information on the relaxation dynamics of the sample as described in the literature^31–33^ and summarized in the Supplementary Information. Examples of model curves fitted to the experimental data are reported as well in Fig. 1 together with the relaxation functions describing the sample dynamics and recalculated using the parameters obtained from the fits. More precisely (see Supplementary Information for details), in TDI experiments with two equal nuclear absorbers (as in the case of the present experiment), the effect of the sample dynamics on the beating pattern can be expressed by a contrast function, *ϕ*′(*q*, *t*). *ϕ*′(*q*, *t*) is proportional to the auto-correlation function of the density fluctuations or normalized intermediate scattering function, *ϕ*(*q*, *t*), via the coefficient $\frac{2}{1+{f}_{{\Delta }_{E}}}$ ^32^. Here *f*_Δ*E*_ is the fraction of the dynamic structure factor (i.e. the Fourier transform of the intermediate scattering function) that overlaps with the incident X-ray beam bandwidth. We describe the contrast function *ϕ*′(*q*, *t*) using the Kohlrausch-Williams-Watts (KWW) model^1^:

1 $${\varphi }^{{\rm{^{\prime} }}}(q,t)={f}_{q}^{{}^{{\rm{^{\prime} }}}}\cdot {e}^{-{(\frac{t}{\tau })}^{{\beta }_{KWW}}},$$

where: *τ* is the relaxation time; *β*_*KWW*_ is the stretching parameter; and ${f}_{q}^{^{\prime} }$ is the contrast of the beating pattern for *t* → 0 and is related to the relaxation strength, *f*_*q*_, via^32^:

2 $${f}_{q}^{^{\prime} }=\frac{2}{1+{f}_{\Delta E}}{f}_{q}.$$

The dielectric loss spectra were analyzed using a simple Lorentzian for the Debye peak, the Fourier transform of the KWW model for the *α* process and the Cole-Cole^25^ expression for the *β*_*JG*_-peak, more details in the Supplementary Information.

### Relaxation map and initial beating pattern contrast

**Figure 2:**
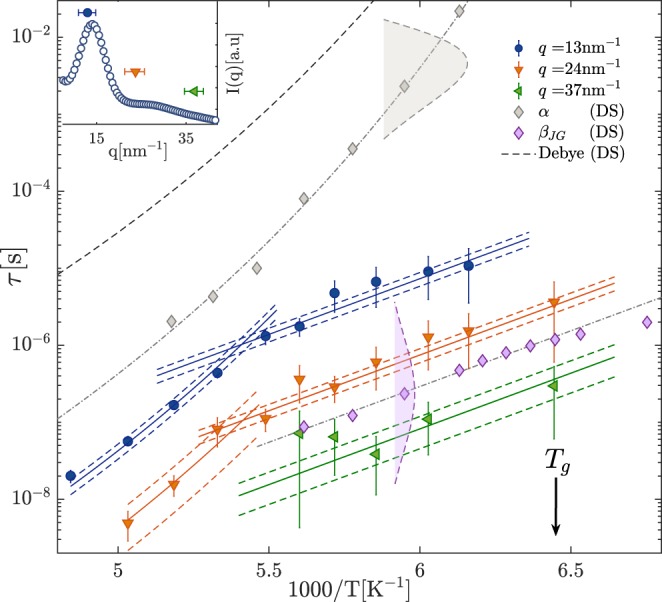
*T*-dependence of the relaxation time measured by TDI at three different *q*-values: *q* = 13 (blue circles), 24 (orange downward-pointing triangles) and 37 nm^−1^ (green left-pointing triangles) and by dielectric spectroscopy (gray diamonds, lilac diamonds). The dotted-dashed lines are fits to the DS data (*α* and *β*_*JG*_ relaxation) whereas the black dashed line refers to the Debye process, detected only by DS. The solid lines are the same fits to the *α* and *β*_*JG*_ relaxations re-scaled to match the TDI data. The dashed lines are the corresponding 95% confidence intervals. The gray and lilac areas delimited by dashed lines at 1000/*T* = 5.95 K^−1^ show the typical shapes of the distributions of relaxation times *G*(ln *τ*) associated to the *α* and *β*_*JG*_ relaxations as extracted from DS data. The base widths of the two areas correspond to the FWHM of the two distributions. Inset: diffuse scattering pattern of 5M2H at *T* = 187.6 K, with the indication of the *q* values and of the corresponding ranges covered in the TDI measurements reported in the main figure.

The relaxation time values obtained by TDI and DS are reported in Fig. 2. The distributions of the natural logarithm of relaxation times *G*(ln *τ*) for the *α* and *β*_*JG*_ processes extracted from the DS measurements are also reported for *T* = 168 K as an example (gray and lilac areas delimited by dashed lines). The *T*-dependencies of the *α* and *β*_*JG*_ relaxation times were obtained fitting the DS measurements (gray diamonds and green upward-pointing triangles, respectively). The *α*-relaxation characteristic time was then modeled using the Vogel-Fulcher-Tammann (VFT) expression *τ* = *τ*_0_ exp(*DT*_0_/(*T* − *T*_0_)), where *τ*_0_, *D* and *T*_0_ are phenomenological parameters. The found values (*D* = 12(2), *τ*_0_ = 10^−12.8(9)^ s and *T*_0_ = 112(5) K) are in agreement with the literature^30^. The *T*-dependence of the *β*_*JG*_-relaxation was instead modeled using the Arrhenius equation: the reduced activation energy found here (*E*/*k*_*B*_ = 3.3(1) × 10^3^ K) agrees with the one reported in ref.^34^. The obtained curves (dash-dotted lines) were then scaled onto the relaxation time data measured by TDI in order to identify unambiguously the *α* and *β*_*JG*_ processes (solid lines in Fig. 2). The dashed-lines correspond to the 95% confidence bands. It clearly emerges from Fig. 2 that the structural relaxation is the dominating process for *T* > *T*_*αβ*_ ≃ 181 K at *q* = 13 and 24 nm^−1^. Regarding *q* = 37 nm^−1^, the dynamics is instead too fast to be detected by TDI in that *T* range. Below *T*_*αβ*_ the *T*-dependence of *τ* changes and it is in close agreement with the one found by DS for the *β*_*JG*_-relaxation.

Below *T*_*αβ*_, that is when the *β*_*JG*_ process separates from the *α* one, and at *q* = 13 and 24 nm^−1^, the characteristic time of the structural relaxation is so slow compared to the time window accessed by TDI that the measured patterns do no show a complete decorrelation. Therefore, TDI is only sensitive to the faster of the two relaxation processes, that is the *β*_*JG*_ one, and their strengths cannot be measured separately. Close to the decoupling temperature *T*_*αβ*_, the *α* and *β*_*JG*_ relaxations coexist in the probed time-window, but the accuracy of our experimental data does not allow us to distinguish between the two. If we consider instead *q* = 37 nm^−1^, it can be noticed that density fluctuations almost completely decorrelate even below *T*_*αβ*_ (see for example Fig. 1(a)): here the *β*_*JG*_ relaxation is the dominant process with a strength much larger than that of the *α* relaxation. It is also interesting to notice that, differently from what observed in previous TDI measurements on OTP^11^ and TDI and neutron scattering measurements on PB^6,16,27^, we observe the *β*_*JG*_ relaxation also at the inter-molecular distance 2*π*/*q*_*max*_ ≃ 4.5 Å, where *q*_*max*_ = 14 Å^−1^ is q-value corresponding to the maximum of the static structure factor, *S*(*q*). We furthermore notice that, consistently to what observed in OTP^11^ and PB^16^, the decoupling of the *β*_*JG*_ from the *α* process takes place at a temperature *T*_*αβ*_ ≃ 181 K which is close to 1.2 *T*_*g*_, the typical value for the mode coupling critical temperature *T*_*c*_.

Looking at Fig. 2(a), it is possible to notice that the time scales for the *α* and *β*_*JG*_ relaxations measured by TDI are less separated than the ones from DS measurements. More specifically, density fluctuations for the *β*_*JG*_ process probed by TDI close to the average inter-molecular distance relax almost two orders of magnitude slower than reorientations: by the time the molecular dipoles loose correlation with respect to the initial orientation, the centers of mass of two neighboring molecules will have barely moved (one relative to the other). Conversely, the *α* relaxation for the density fluctuations is always faster than that appearing in dipole reorientations.

It has to be underlined that the Debye relaxation^28,34^, much slower than the *α* one, was detected only by DS and in Fig. 2 only its characteristic timescale is reported for the sake of clarity. Indeed, the Debye process, despite being the dominant one in DS data, is related to fluctuations of the end-to-end dipole moment of transient supramolecular structures and has therefore little or no signature in the spectra of other experimental techniques such as depolarized light-scattering^34,35^, mechanical spectroscopy^36^, triplet solvatation state with mechanical probes^37^ as well as in differential scanning calorimetry^38^. Concerning microscopic density fluctuations, neutron scattering experiments on 1-propanol, another monohydroxyl alcohol showing a similar Debye feature in DS spectra, have revealed that at the maximum of *S*(*q*) the relaxation dynamics is dominated by the *α* process^39^. In agreement with these results, in the present experiment no sign of a process slower than the *α* relaxation was detected. This is for example very clear in the TDI beating pattern at *T* = 206.8 K and *q* = 13 nm^−1^ reported in Fig. 2(b), where a complete decorrelation can be observed following the structural process, implying that the Debye process has a negligible strength in the density-density correlation function of 5M2H at the probed length-scales and time-scales.

**Figure 3:**
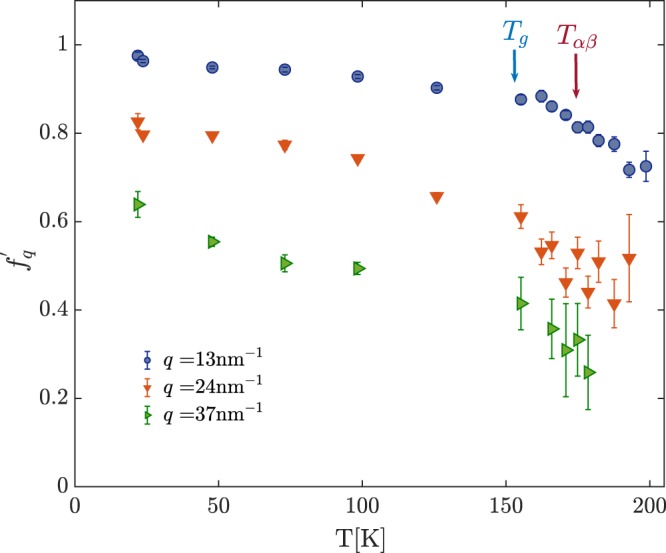
*T*-dependence of the initial contrast of the beating pattern, ${f}_{q}^{^{\prime} }$, measured at three different exchanged wave-vectors: 13 (blue circles), 24 (downward-pointing orange triangles) and 37 nm^−1^ (left pointing green triangles).

The *T*-dependence of the initial contrast of the beating pattern ${f}_{q}^{^{\prime} }$ corresponding to the *τ* values of Fig. 2 is shown in Fig. 3. There, also the ${f}_{q}^{^{\prime} }$ values measured below *T*_*g*_, where the decorrelation of density fluctuations is too slow to be probed by TDI, are reported. As already stressed before, the TDI measurements detect at each *T* and *q* only one relaxation process, and ${f}_{q}^{^{\prime} }$ accounts for the total strength of the *α* and *β*_*JG*_ processes. A clear change in the *T*-dependence of ${f}_{q}^{^{\prime} }$ can be observed for all *q* values around *T*_*g*_ = 154 K, as expected when the glass transition occurs, whereas no discontinuity is observed at *T*_*αβ*_ = 181 K. This is a well-know result from DS and depolarized dynamic light scattering measurements, that is when the *β*_*JG*_ relaxation separates from the structural one no discontinuity in the total strength is detected^34,35^. It is interesting to notice that also at 37 nm^−1^, where the *β*_*JG*_ relaxation is the dominant relaxation channel, a clear change in the temperature dependence of the contrast is observed at *T*_*g*_, thus highlighting the sensitivity of the *β*_*JG*_ relaxation to the glass-transition, as already shown for the dielectric strength of the *β*_*JG*_ process at *T*_*g*_^1^. More information on the temperature dependence of ${f}_{q}^{^{\prime} }$ is reported in the Supplementary Information.

### Wave-number dependence of the relaxation parameters

**Figure 4:**
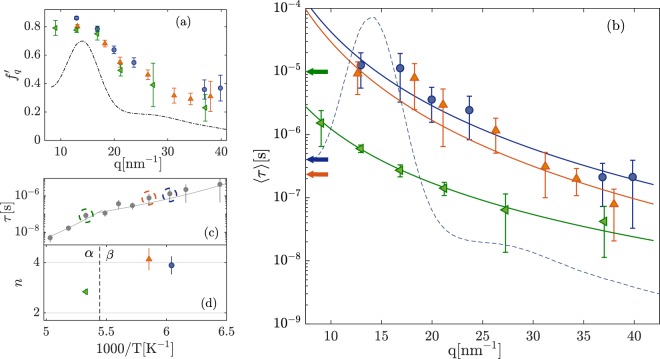
(**a**) Wave-number (*q*) dependence of the initial beating pattern contrast. The diffuse scattering pattern measured at *T* = 187.6 K is reported for sake of comparison. (**b**) *q*-dependence of the mean relaxation time at three different temperatures *T* = 187.6 K (green left-pointing triangles), 170.4 K (orange upward-pointing triangles) and 165.5 K (blue dots). The diffuse scattering pattern measured at *T* = 187.6 K is reported once more to facilitate comparison. The horizontal arrows point to the corresponding relaxation times measured by dielectric spectroscopy. The solid lines are power law fits to the data using the expression: *τ* ∝ *q*^−*n*^. (**c**) Temperature dependence of the relaxation time at *q* = 24 nm^−1^, as in Fig. 2. The circles indicate the temperatures where the *q*-dependencies were studied. (**d**) Power-law exponents, *n*, from the fitted model curves shown in (**b**), as a function of the inverse temperature.

Three temperatures were selected for a more detailed investigation of the *q*-dependence of the main parameters of the two relaxation processes (*α* and *β*_*JG*_): one in the range where only the structural relaxation is present (*T* = 187.6 K) and the other two (*T* = 170.4 and 165.5 K) where only the *β*_*JG*_ relaxation is detected. The corresponding results are reported in Fig. 4. The *q*-dependence of the beating pattern contrast, ${f}_{q}^{^{\prime} }$, reported in Fig. 4(a), shows a shallow oscillation in phase with the main peak of the total scattered intensity of the sample. This is already well known from both simulations^40^ and experiments^41^ for what concerns the structural relaxation, and is an indication of the sensitivity of the *α* relaxation to the structure. At *q*-values higher than *q*_*max*_ in the range 31–40 nm^−1^ and for 181 K > *T* > 165 K, we observe that density fluctuations decorrelate completely within the experimental time-window and therefore the *β*_*JG*_ process is by far the dominating relaxation. This allows us to estimate in that range ${f}_{q}^{^{\prime} }$ for the *β*_*JG*_ relaxation. Averaging the beating pattern contrast in the aforementioned *T* and *q* interval, we get a value of 〈${f}_{q}^{{}^{{\rm{^{\prime} }}}}$〉_*T*,*q*_ = 0.31(2). In order to estimate the strength of the process we need to account for the correction factor reported in Eq. 2. This factor depends on the experimental set-up and on material properties, and has been estimated in^32^ for glycerol in a comparable *q* and *τ* range. If this value for the correction factor is used for our case here, we can estimate the *β*_*JG*_ strength to be ≃0.25, while the strength of the *α* process is negligible. This implies that at the intra-molecular scale, if we imagine to take a snapshot of the system, about one fourth of the molecules relaxes, in average, via the *β*_*JG*_ relaxation, whereas faster processes (fast relaxations, vibrations) account for the remaining strength. Of course, when followed over a long enough time period, all molecules will participate, in average, to the *β*_*JG*_ process. To the best of our knowledge, this is the first estimation of the strength of the *β*_*JG*_ relaxation process probed via density fluctuations, though at the moment this is limited to a single average value in the aforementioned *T*-range.

We now consider the *q*-dependence of the mean relaxation time 〈*τ*〉, calculated using the well-known relation $\tau =\tau {\Gamma }_{E}(\frac{1}{{\beta }_{KWW}})/{\beta }_{KWW}$, where Γ_*E*_ is the Euler gamma function. The *q*-dependence of 〈*τ*〉 is reported in Fig. 4(b), along with the 〈*τ*〉_*DS*_ obtained from the DS measurements at the same temperatures and for both the *α* and *β*_*JG*_ relaxations (arrows with the same color as for the corresponding TDI values). In particular, 〈*τ*〉_*DS*_ for the *α*-process is the mean relaxation time, since the corresponding peak in the dielectric loss-spectrum was fitted using the KWW model as for the TDI data; 〈*τ*〉_*DS*_ for the *β*_*JG*_ relaxation, which was fitted using the Cole-Cole model in the DS spectra, was instead obtained (i) first transforming the relaxation parameters into the ones for the KWW model^42^ and then (ii) calculating the mean as for the *α*-relaxation. The *q*-dependence of 〈*τ*〉 was fitted using a simple power-law: 〈*τ*〉 ∝ *q*^−*n*^. Within the accuracy of our measurements, this simple model describes well the data at all the investigated *q*’s. A super-quadratic, i.e. *n* > 2, dependence is observed for both relaxations with *n* = 2.8(1) for the *α* and 4.1(4) and 3.9(3) for the *β*_*JG*_ relaxation at the two investigated temperatures (see Fig. 4(d)).

## Discussion

A more-than quadratic *q*-dependence for the *α*-relaxation indicates that, close to *T*_*αβ*_, the *α* process has a non-diffusive (‘sub-diffusive’) character. The same is true even more for the *β*_*JG*_-relaxation, given that *n* ≃ 4. It is interesting to observe that a similar coefficient has been reported for OTP^17^, a van der Waals glass-former, below the glass transition temperature. Such a strong *q*-dependence is an indication that the *β*_*JG*_-process is characterized by an anomalous, restricted dynamics at this length-scale.

We can learn more on the microscopic mechanisms associated to the *β*_*JG*_ relaxation from a detailed comparison between the DS and the TDI data. Relating density fluctuations results to dipole reorientations is not always straightforward, as exemplified by the case of PB. In that case the characteristic time of the *β*_*JG*_ relaxation measured by DS is two orders of magnitude slower than that measured by neutron spin echo (NSE)^6^. A possible interpretation of this large difference could be given only recurring to simulations which suggested NSE and DS to be sensitive to the motion of different units in PB^43^. In the present case of 5M2H this comparison is made easier by the fact that we are dealing with a small molecule and that the *β*_*JG*_ relaxation time has a strong pressure dependence^30^, evidence that it is not of intra-molecular origin. This is also consistent with the fact that the timescale of the *β*_*JG*_ relaxation probed by DS in 5M2H^29^ is approximately matched by the prediction of the coupling model for the precursor of the cooperative *α*-relaxation, as previously obtained in a number of glass-formers^1,5^. Moreover, we can take advantage of the observation that, below the decoupling temperature *T*_*αβ*_, the relaxation times characteristic of the density-density correlation function probed by TDI in a *q*-range covering both the inter-molecular and the intra-molecular length-scale have the same temperature dependence of the DS characteristic time, see Fig. 2. This observation has a few relevant consequences.

(i) The density-density correlation functions at *q*’s in the range 13–37 nm^−1^ couple to the same relaxation and in the same way. This implies that, differently from the case of PB previously referred to, the density-density correlation function just reflects the CM dynamics at different *q*’s. In other terms, we can approximate 5M2H as being a rigid molecule in the *q* and time range of interest here. Would this not be the case, we would observe some kind of intra-molecular dynamics and therefore expect a different *T*-dependence for the characteristic time probed at different *q*’s. This conclusion is consistent with the observed equivalence between DS and depolarized dynamic light scattering data in the *β*_*JG*_ range of 5M2H^34^ which signals that the symmetry axis of the polarizability lying along the alkyl chain and the dipole moment, oriented along the OH-bond, maintain a fixed relative orientation angle.
(ii) The microscopic CM (translational) dynamics at different *q*’s and the rotational dynamics are coupled to the same relaxation and are therefore coupled one to the other.
(iii) If the *β*_*JG*_ relaxation is characterized by a specific length-scale, in view of the strong roto-translational coupling discussed above, we should expect to measure the same relaxation time for molecular dipole reorientations and for CM translations if the latter ones are probed at that length-scale. Namely, in view of the roto-translation coupling discussed above, the *q* value at which we find a match of the characteristic times obtained by DS and TDI gives us precise information on the characteristic length-scale of the *β*_*JG*_ relaxation. Figure 2 shows that our TDI data cover in fact this interesting condition: at high *q* values (*q* > 28 nm^−1^) density fluctuations decorrelate on a timescale that is faster than or similar to that of the permanent dipole reorientations, whereas it becomes clearly slower on approaching *q*-values that correspond to the average inter-molecular distance. The characteristic time for the *β*_*JG*_ process matches the one measured by DS for *q*_*JG*_ = 3.3 Å^−1^. At that *q* value, relative CM translations of a pair of molecules decorrelate after a relative motion 1/*q*_*JG*_ = 0.3 Å. This is then the characteristic (most likely) length scale for CM translations within the *β*_*JG*_ process.
(iv) Figure 2 also gives evidence of the *β*_*JG*_ relaxation up to *q*_*max*_, the maximum of the diffuse scattering intensity. This conclusion is new with respect to what was concluded in previous measurements^11,16^, and implies that, while the *β*_*JG*_ process has a small characteristic length-scale of the order of 0.3 Å, it includes molecular rearrangements that extend at least up to inter-molecular distances.

**Figure 5:**
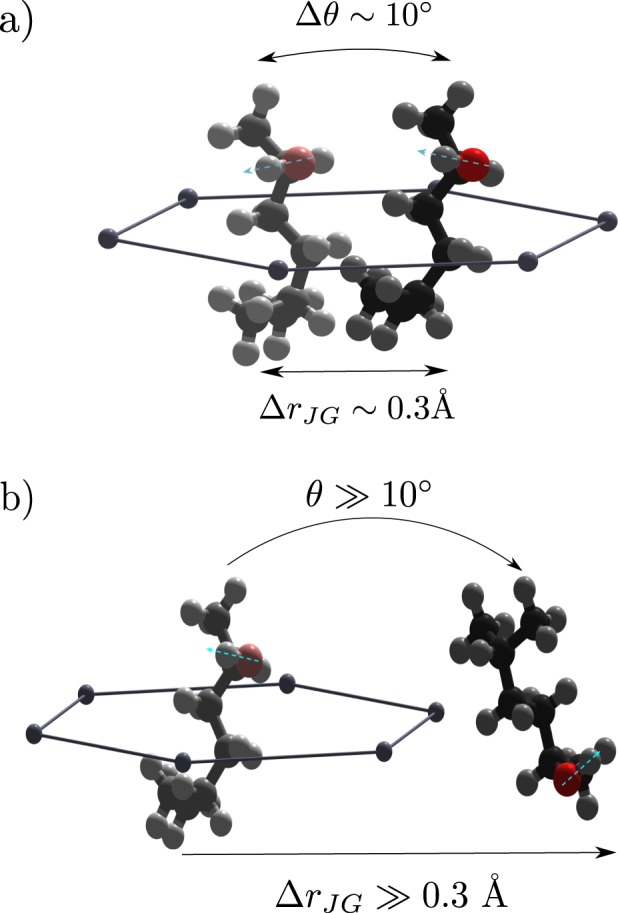
Schematic illustration of the rearrangement of a 5-methyl-2-hexanol molecule undergoing the Johari-Goldstein relaxation. The most likely process consists of restricted translational and reorientational dynamics occurring in the local environment defined by the first neighbors (**a**). Large spatial and angular excursions, responsible for cage-escape events, are also present (**b**). The cyan dashed lines in (**a**,**b**) represent the orientation of the dipole moment of the molecule, along the O-H bond.

When we put together the information collected here on the *β*_*JG*_ process with that available in the literature and mainly related to the reorientational dynamics, the following picture comes out. Reorientations take place through large-angles rearrangements at high temperatures^26^, and become more restricted as the glass-transition temperature is approached. At *T*_*g*_ the amplitude of these reorientations is of the order of 10° ^7^, the details depending of course on the molecular structure. In addition to these small-angle reorientations, in the same temperature range a fraction of large-angle (≫10°) reorientations also takes place. The existence of the latter ones has been observed in numerical simulation studies of molecular and polymeric model systems^20^; however, these simulations refer to systems quenched to temperatures well below *T*_*g*_ and therefore the comparison to the present TDI results is not straightforward. Experimentally, large angle reorientations can be deduced e.g. from combined depolarized dynamic light scattering and DS measurements on 5M2H and 1-propanol^34,35^ which show that the correlation functions relative to the *β*_*JG*_ process probed by the two techniques are equivalent for both mono-alcohols. This strict equivalence can in fact be explained only if large-angle reorientations are present, at least for a fraction of the molecules, and even at *T*_*g*_. The strong roto-translational coupling here reported suggests a parallel scenario for the CM translations. Specifically, we have seen how the *β*_*JG*_ process is characterized by CM rearrangements of the order of 10% of the inter-molecular distance that take place within the cage formed by the first neighbours (see Fig. 5(a)). This is consistent with the recent neutron scattering results on propylene carbonate^19^. In addition, and as for the molecular rotations, a fraction of rearrangements will be characterized by a much longer length-scale. We can in fact say that the characteristic lengths distribution for the *β*_*JG*_ process extends at least up to the inter-molecular distance, and therefore that while this process is mainly local, it does have tails extending to longer distances. This is consistent with the fact that the distribution of relaxation times *G*(ln *τ*) extracted from DS measurements for the *β*_*JG*_ relaxation is characterized by very long tails extending over more than two decades (see Fig. 2) and reaching the characteristic timescales for density fluctuations at the inter-molecular length scale. In other terms, the restricted dynamics for both CM translations and reorientations will occasionally be interrupted by longer ranged displacements and large-angle reorientations. It is interesting that our results obtained for a hydrogen-bonded glass-former are consistent with recent simulations for model metallic glasses^18,21^ suggesting that the *β*_*JG*_ relaxation takes place via cooperative rearrangements of string-like clusters. In fact, our results clarify that the *β*_*JG*_ relaxation process probed by the microscopic density correlation function is dominated by spatial fluctuations of a molecule within the cage formed by the nearest neighbours, i.e. by rattling motions within the cage. At the same time, we know that part of the relaxation strength, corresponding to longer relaxation times, is related to larger spatial fluctuations extending at least up to the intermolecular distance. It is this fraction of motions that we associate to escape processes from the cage (see Fig. 5(b)) and that are likely related to the string-like dynamics elucidated in numerical simulations^18,21^.

We can thus visualize the *β*_*JG*_-relaxation, above the glass-transition temperature where its characteristic time is of the order of 100 ns (*T* < 181 K), as a local process, where, in turn, one out of four molecules shows a restricted dynamics within the cage of its neighbours and is characterized by rearrangements of about 10% of the intermolecular distance. In fact, the *β*_*JG*_ relaxation dominates at intramolecular distances (31 nm^−1^ < *q* < 40 nm^−1^), where ≃25% of the molecules participate, in average, to the *β*_*JG*_-process and the *α*-relaxation is negligible, whereas the remaining molecules are involved in fast relaxation processes and vibrations. However, this is not the whole story. We know in fact directly from our measurements that molecular rearrangements within the *β*_*JG*_ process must extend at least up to the inter-molecular distance, and are therefore related to cage-breaking processes. It would be clearly interesting to probe directly this cooperative part of the *β*_*JG*_ process. For what concerns the CM dynamics, this implies extending the present studies towards lower *q*-values. This is experimentally challenging, since it implies looking at the longer times within the distribution of relaxation times for the *β*_*JG*_ process and at small relaxation strengths. We plan to move in this direction in the near future.

## Methods

### Nuclear *γ*-resonance time-domain interferometry measurements

The nuclear *γ*-resonance TDI experiment, an optimized implementation of what is originally described in^31^, was carried out at the nuclear resonance beamline ID18^44^ of the European Synchrotron Radiation Facility (ESRF) in Grenoble (France). The incident X-ray radiation, characterized by a bandwidth of 2.5 meV at the energy of the first nuclear transition of ^57^Fe (14.412 keV), was selected using a high resolution monochromator. Two single-line ^57^*Fe*-containing absorbers were installed upstream of the sample, on the incoming beam, and downstream of the sample, on the scattered beam, to provide the probe and reference beam of a time-domain interferometer. In order to obtain different excitation energies, the probe absorber was mounted on a velocity transducer and driven at the constant velocity *v* = 10 mm/s with a relative accuracy better than 0.1%. Three avalanche photodiode (APD) detectors were used to simultaneously collect the photons quasi-elastically scattered by the sample at three different scattering vectors *q* = 2*k*_0_*sin*(*θ*/2), where *θ* is the scattering angle and *k*_0_ = 73 nm^−1^ is the wave-vector of the nuclear fluorescence from the first excited state of ^57^*Fe*. The setup was designed to span the *q*-range 9–40 nm^−1^. Further details can be found in the Supplementary Information.

### Sample

The 5M2H sample was purchased from Sigma Aldrich (purity ≃ 98%) and used as received. 5M2H was chosen since investigations of the *β*_*JG*_ relaxation at the microscopic scale have not yet been reported for hydrogen-bonded glass-formers. In particular 5M2H displays a *β*_*JG*_ relaxation strongly separated in timescale from the structural one above *T*_*g*_^30,34^. The temperature of the sample was controlled using a He-flow cryostat with ±0.1 K stability.

### Dielectric spectroscopy measurements

The complex permittivity of the sample was measured in the range 10 mHz–10 MHz using a lumped impedance technique and the Novocontrol Alpha-Analyzer, whereas in the range 1 MHz–3 GHz using the coaxial reflectometric technique^45^ employing the Agilent 8753ES Network Analyzer. The dielectric cell consisted in parallel plate capacitors separated by silica spacers and filled by the sample in the liquid state. The temperature of the sample was controlled using a dry nitrogen-flow Quatro cryostat with a temperature accuracy of better than 0.1 K.

## Supplementary information

Supplementary information

## Data Availability

Data are available from the corresponding authors upon reasonable request.

## References

[1] Ngai, K. L. Relaxation and Diffusion in Complex Systems. *Springer, New York* (2011).

[2] Johari GP, Goldstein M (1970). Viscous Liquids and the Glass Transition. II. Secondary Relaxations in Glasses of Rigid Molecules. J. Chem. Phys.

[3] Ngai KL (1998). Relation between some secondary relaxations and the *α*-relaxations in glass-forming materials according to the coupling model. J. Chem. Phys..

[4] Paluch M, Ngai KL (2004). Classification of secondary relaxation in glass-formers based on dynamic properties. J. Chem. Phys..

[5] Capaccioli S, Paluch M, Prevosto D, Wang L-M, Ngai KL (2012). Many-body nature of relaxation processes in glass-forming systems. J. Phys. Chem. Lett..

[6] Arbe A (1996). Study of the dynamic structure factor in the *β*-relaxation regime of polybutadiene. Phys. Rev. Lett..

[7] Vogel M, Rössler EA (2001). Slow *β* process in simple organic glass formers studied by one- and two-dimensional ^2^*H* nuclear magnetic resonance. I. J. Chem. Phys..

[8] Tanaka H (2005). Two-order-parameter model of the liquid–glass transition. III. Universal patterns of relaxations in glass-forming liquids. J. Non–Cryst. Solids.

[9] Richert R, Samwer K (2007). Enhanced diffusivity in supercooled liquids. New J. Phys..

[10] Stevenson JD, Wolynes PG (2010). A universal origin for secondary relaxations in supercooled liquids and structural glasses. Nat. Phys..

[11] Saito M (2012). Slow Processes in Supercooled o-Terphenyl: Relaxation and Decoupling. Phys. Rev. Lett..

[12] Yu HB, Samwer K, Wu Y, Wang WH (2012). Correlation between *β*-relaxation and self-diffusion of the smallest constituting atoms in metallic glasses. Phys. Rev. Lett..

[13] Yu HB, Samwer K, Wang WH, Bai HY (2013). Chemical influence on *β*-relaxations and the formation of molecule-like metallic glasses. Nat. Commun..

[14] Micko B, Tschirwitz C, Rössler EA (2013). Secondary relaxation processes in binary glass formers: Emergence of “islands of rigidity”. J. Chem. Phys..

[15] Cicerone MT, Zhong Q, Tyagi M (2014). Picosecond Dynamic Heterogeneity, Hopping, and Johari-Goldstein Relaxation in Glass-Forming Liquids. Phys. Rev. Lett..

[16] Kanaya T, Inoue R, Saito M, Seto M, Yoda Y (2014). Relaxation transition in glass-forming polybutadiene as revealed by nuclear resonance X-ray scattering. J. Chem. Phys..

[17] Saito M (2014). Slow dynamics of supercooled liquid revealed by Rayleigh scattering of Mössbauer radiation method in time domain. Hyp. Int..

[18] Yu HB, Richert R, Samwer K (2017). Structural rearrangements governing Johari-Goldstein relaxations in metallic glasses. Sci. Adv..

[19] Cicerone MT, Tyagi M (2017). Metabasin transitions are Johari-Goldstein relaxation events. J. Chem. Phys..

[20] Fragiadakis D, Roland CM (2017). Participation in the Johari–Goldstein process: molecular liquids versus polymers. Macromolecules.

[21] Yu H-B (2018). Fundamental Link between *β* Relaxation, Excess Wings, and Cage-Breaking in Metallic Glasses. J. Phys. Chem. Lett..

[22] Yu HB (2012). Tensile Plasticity in Metallic Glasses with Pronounced *β* Relaxations. Phys. Rev. Lett..

[23] Ngai KL, Wang L-M, Liu E, Wang WH (2014). Microscopic dynamics perspective on the relationship between Poisson’s ratio and ductility of metallic glasses. J. Chem. Phys..

[24] Bhattacharya S, Suryanarayanan R (2009). Local mobility in amorphous pharmaceuticals—characterization and implications on stability. J. Pharm. Sci..

[25] Kremer, F. & Loidl, A. Advances in Dielectrics: The Scaling of Relaxation Processes, *Springer International Publishing* (2018).

[26] Kudlik A, Benkhof S, Blochowicz T, Tschirwitz C, Rössler EA (1999). The dielectric response of simple organic glass formers. J. Mol. Struct..

[27] Richter D, Zorn R, Farago B, Frick B, Fetters LJ (1992). Decoupling of time scales of motion in polybutadiene close to the glass transition. Phys. Rev. Lett..

[28] Böhmer R (2014). Structure and dynamics of monohydroxy alcohols - Milestones towards their microscopic understanding, 100 years after Debye. Physics Reports.

[29] Ngai K-L, Wang L-M (2019). Relations between the structural *α*-relaxation and the Johari-Goldstein *β*-Relaxation in two monohydroxyl alcohols: 1-propanol and 5-methyl-2-hexanol. J. Phys. Chem. B..

[30] Pawlus S, Paluch M, Nagaraj M, Vij JK (2011). Effect of high hydrostatic pressure on the dielectric relaxation in a non-crystallizable monohydroxy alcohol in its supercooled liquid and glassy states. J. Chem. Phys..

[31] Baron AQR (1997). Quasielastic scattering of synchrotron radiation by time domain interferometry. Phys. Rev. Lett..

[32] Saito M, Masuda R, Yoda Y, Seto M (2017). Synchrotron radiation-based quasi-elastic scattering using time-domain interferometry with multi-line gamma rays. Sci. Rep..

[33] Caporaletti F, Chumakov AI, Rüffer R, Monaco G (2017). A new experimental scheme for nuclear *γ*-resonance time-domain interferometry. Rev. Sci. Instrum..

[34] Gabriel J, Pabst F, Helbling A, Böhmer T, Blochowicz T (2018). On the Nature of the Debye-Process in Monohydroxy Alcohols: 5-Methyl-2-Hexanol Investigated by Depolarized Light Scattering and Dielectric Spectroscopy. Phys. Rev. Lett..

[35] Gabriel J, Pabst F, Blochowicz T (2017). Debye Process and *β*-Relaxation in 1-Propanol Probed by Dielectric Spectroscopy and Depolarized Dynamic Light Scattering. J. Phys. Chem. B.

[36] Jakobsen B, Maggi C, Christensen T, Dyre JC (2008). Investigation of the shear-mechanical and dielectric relaxation processes in two monoalcohols close to the glass transition. J. Chem. Phys..

[37] Wendt H, Richert R (1998). Purely mechanical solvation dynamics in supercooled liquids: The *S*_0_ ← *T*_1_ (0-0) transition of naphthalene. J. Phys. Chem. A.

[38] Huth H, Wang L-M, Schick C, Richert R (2007). Comparing calorimetric and dielectric polarization modes in viscous 2-ethyl-1-hexanol. J. Chem. Phys..

[39] Sillrén P (2014). Liquid 1-propanol studied by neutron scattering, near-infrared, and dielectric spectroscopy. J. Chem. Phys..

[40] Sciortino F, Fabbian L, Chen S-H, Tartaglia P (1997). Supercooled water and the kinetic glass transition. II. Collective dynamics. Phys. Rev. E.

[41] Tölle A (2001). Reports on progress in physics neutron scattering studies of the model glass former ortho-terphenyl. Rep. Prog. Phys..

[42] Alvarez F, Alegria A, Colmenero A (1991). Relationship between the time-domain Kohlrausch-Williams-Watts and frequency-domain Havriliak-Negami relaxation functions. Phys. Rev. B.

[43] Narros A, Arbe A, Alvarez F, Colmenero J, Richter D (2008). Atomic motions in the *αβ*-merging region of 1, 4-polybutadiene: A molecular dynamics simulation study. J. Chem. Phys..

[44] Rüffer R, Chumakov AI (1996). Nuclear resonance beamline at ESRF. Hyp. Int..

[45] Wei Y, Sridhar S (1989). Technique for measuring the frequency–dependent complex dielectric constants of liquids up to 20 GHz. Rev. Sci. Instrum..

