# Supplementary material for: A microscopic look at the Johari-Goldstein relaxation in a hydrogen-bonded glass-former
Source: Sci Rep. 2019 Oct 4;9:14319. doi: 10.1038/s41598-019-50824-7 (PMC6778113; doi:10.1038/s41598-019-50824-7)
Supplement: Supplementary file 1 — Supplementary information [file 41598_2019_50824_MOESM1_ESM.pdf]

# Supplementary Information

# A microscopic look at the Johari-Goldstein relaxation in a hydrogen-bonded glass-former

F. Caporaletti[1], S. Capaccioli[2,3], S. Valenti[4], M. Mikolasek[5], A. I. Chumakov[5,6] and G. Monaco[1].

[1]Dipartimento di Fisica, Università di Trento, I-38123 Povo (Trento), Italy.

[2]Dipartimento di Fisica, Universita di Pisa, Largo Bruno Pontecorvo 3, I-56127 Pisa, Italy.

[3]CNR-IPCF, Largo Bruno Pontecorvo 3, I-56127 Pisa, Italy.

[4]Grup de Caracteritzaciò de Materials, Department of Physics, Universitat Politècnica de Catalunya, EEBE, Av. Eduard Maristany 10-14, E-08019 Barcelona, Spain.

[5]ESRF-The European Synchrotron, CS40220, 38043 Grenoble Cedex 9, France.

[6]National Research Center ``Kurchatov Institute", 123182 Moscow, Russia

## S1: Nuclear gamma-resonance time interformetry

A sketch of the nuclear nuclear γ-resonance time-domain interferometer (TDI) setup employed for the experiment here discussed is shown in Fig. S1. The synchrotron radiation used in the TDI experiment was characterized by a bandwidth of 2.5 meV, see Fig. S2, at the energy of the first nuclear transition of $^{57}Fe$ ($E_0$=14.412 keV) and it was selected using, in cascade, a high heat load monochromator and a high resolution one. The high heat-load monochomator consisted of two separate Si crystals in (111) reflection [S1]. The high-resolution monochromator, instead, was realized by a combination of four asymmetrically cut Si crystals similar to that described in [S2]. Two single-line absorbers containing $^{57}Fe$, made up of pellets of a powder of $K_2Mg^{57}Fe[CN]_6$ (1 mg of $^{57}Fe$ per cm$^2$), were used to provide both the (upstream) probe and (downstream) reference beams. In order to achieve different excitation energies, the probe absorber was mounted on a velocity transducer and driven at the constant velocity of v=10 mm/s with a relative accuracy better than 0.1%.

Three double avalanche photodiode (APD) detectors (EG&G Optoelectronics, 10x10 mm$^2$ active area), placed at a distance $d = 80$ mm from the sample and characterized by an efficiency of 66%, were used to simultaneously collect the photons quasi-elastically scattered by the sample at three different scattering vectors $q = 2k_0 sin\left(\frac{\theta}{2}\right)$, where $\theta$ is the scattering angle and $k_0 = 73$ nm$^{-1}$ is the wave-vector of the nuclear fluorescence from the first excited state of $^{57}Fe$. A fourth APD detector monitored the resonant transmitted intensity (nuclear forward scattering) and thus the stability of the experimental set-up during the measurements.

For what concerns the sample-environment, a copper cell of length $l$=13 mm with 50 $\mu$m thick kapton windows was used as sample holder. $l$ was chosen in order to match the attenuation length of 5-methyl-2-hexanol at 14.412 keV. The temperature of the sample was controlled using a He-flow cryostat ( $\pm$ 0.1 K stability) in order to reduce as much as possible mechanical vibrations.

## S2: Scattered wave-number resolution

The scattering vector ($q$) resolution of the experimental set-up described above is controlled by three main parameters: i) the synchrotron radiation spot size at the sample, ii) the finite length of the sample and iii) the solid angle intercepted by the Avalanche Photo-Diode (APD) detector. The final distributions of $q$'s is given by the convolution of these three contributions. The parameters i) and ii) are set by the X-rays optics and by the attenuation length of the sample at the X-ray energy used for the experiment; parameter iii) is fixed by the

geometry which was in turn designed in order to achieve a total $q$-resolution of $\simeq \pm 2$ nm$^{-1}$ at all the studied scattering angles.

## S3: Data treatment

### S3.1 Broadband dielectric spectroscopy data.

The real and imaginary part of the measured permittivity function, $\epsilon'(\nu)$ and $\epsilon''(\nu)$, where $\nu$ is the frequency, were simultaneously fitted. The function used for the fits is:

$$\epsilon(\nu) = \frac{\Delta\epsilon_D}{1 + j\,2\pi\nu\tau_D} + \Delta\epsilon_\alpha\,\mathcal{L}_{j2\pi\nu}\left\{-\frac{d}{dt}\,exp\left[-\left(\frac{t}{\tau_\alpha}\right)^{\beta_{KWW}}\right]\right\} + \frac{\Delta\epsilon_{\beta_{JG}}}{1 + \left(j\,2\pi\nu\tau_\beta\right)^a} + \frac{\sigma}{j2\pi\nu\epsilon_0} + \epsilon_\infty\,, \quad (S1)$$

where the first Lorentzian accounts for the Debye relaxation; the second Kohlrausch-Williams-Watts (KWW) term for the $\alpha$-relaxation; the third one, that is the Cole-Cole function, for the $\beta_{JG}$-relaxation; the fourth for the d.c. conductivity contribution; and the last is the induced polarization dielectric constant. $\Delta\epsilon_{D,\alpha,\beta_{JG}}$ are the dielectric relaxation strengths of the different processes. $\mathcal{L}_{j2\pi\nu}\{\,\}$ is the Laplace transform evaluated at $j2\pi\nu$.

More details on the model used here can be found in [S3].

An example of a dielectric spectroscopy loss spectrum of 5-methyl-2-hexanol at T=163 K is reported in Fig. S3 along with the curve obtained from the fitting procedure (blue solid line) and the individual contributions of the Debye (black solid line), $\alpha$ (red solid line) and $\beta_{JG}$ (green solid line) relaxations.

### S3.2 Nuclear $\gamma$-resonance time-domain interferometry data

In a typical TDI experiment, two Mössbauer absorbers (usually containing $^{57}$Fe) with different energy spectra are placed upstream and downstream of the sample [31]. The nuclear fluorescence following their excitation by a pulse of synchrotron radiation is used to generate both the probe and reference $\gamma$-rays for the TDI. The time evolution of the reference and probe beams at the detector position is characterized by an interference beating pattern which is modulated by the normalized correlation function $\phi_q(t)$ of the density fluctuations, also known as intermediate scattering function [11,31-33,S4]:

$$\phi_q\,(t) = \frac{\langle\,\delta\rho_q^*\,(0)\delta\rho_q\,(t)\rangle}{\left\langle\,\left|\delta\rho_q(0)\right|^2\,\right\rangle}\,, \quad (S2)$$

where $\delta\rho_q(t)$ is the Fourier component of wave-vector $q$ of the fluctuation of the microscopic density $\rho(r,t)$ and $\langle\cdot\rangle$ denotes an ensemble average.

$\phi_q(t)$ is related to the dynamic structure factor $S(\boldsymbol{q},\omega)$ via:

$$\phi_q(t) = \frac{\int_{-\infty}^{+\infty} S(q,\omega)e^{-i\omega t}d\omega}{\int_{-\infty}^{+\infty} S(q,\omega)d\omega}\,, \quad (S3)$$

The TDI beating patterns collected during the experiment have been treated according to the models already reported in the literature [11,31-33,S4]. More specifically, the time-domain evolution of the TDI interferogram, in the hypothesis of negligible radiative coupling between the reference and probe absorber, is described by [32]:

$$I(q,t) \propto \left|G\left(T_{eff}^{1},t\right)\right|^{2} + \left|G\left(T_{eff}^{2},t\right)\right|^{2} \cdot f_{\Delta E} + 2Re\left\{G\left(T_{eff}^{1},t\right)G^{*}\left(T_{eff}^{2},t\right)\right\}\phi_{q}(t) \quad (S4)\,.$$

Here $G\left(T_{eff}^{1,2},t\right)$ are the responses of the upstream and downstream absorbers, with effective thickness $T_{eff}^{1,2}$. $T_{eff}^{1,2}$ is defined as $n\sigma L$, where $n$ is the surface density of resonant nuclei in the absorber, $\sigma$ is the resonant cross-section and $L$ is the thickness of the sample. $f_{\Delta E}$ is the fraction of the dynamic structure factor overlapping with the bandwidth of the incident synchrotron radiation [32]:

$$f_{\Delta E} = \frac{\int_{-\infty}^{+\infty} S(\boldsymbol{q},\omega)I(-\omega)d\omega}{\int_{-\infty}^{+\infty} S(\boldsymbol{q},\omega)d\omega}\,, \qquad (S5)$$

where $I(-\omega)$ is the area-normalized energy spectrum of the incident synchrotron radiation pulse shown in Fig. S2.

The probe and reference beams of the TDI were implemented using two single-line absorbers consisting of pellets produced pressing a $K_2Mg^{57}Fe[CN]_6$ powder, and resulted to have a nearly identical effective thickness $T_{eff}^{1,2} = 9.8$. As already said, the probe upstream absorber was driven at constant velocity $v = 10$mm/s; a shift of $\hbar\Omega$=105(4) $\Gamma_0$, where $\Gamma_0$ is the natural linewidth of the first excited state of $^{57}$Fe, was then obtained. In this scheme, similar to the ones reported in [31,S4], the responses of the probe and reference absorbers are given by:

$$G_{1,2}\left(t,T_{eff}\right) = -\frac{T_{eff}}{2}e^{-i\omega_{1,2}t-\frac{t}{2\tau}}\left[J_1\frac{\left(\sqrt{\frac{T_{eff}t}{\tau}}\right)}{\sqrt{\frac{T_{eff}t}{\tau}}}\right] = R\left(t,T_{eff}\right)e^{-i\omega_{1,2}t}\,, \qquad (S6)$$

where $\omega_{1,2}$ are the resonance energies of the absorbers, $\tau = \hbar/\Gamma_0$ is the lifetime of the excited nuclear resonance, $J_1$ is the Bessel function of first order and first kind. All of the terms not involving the resonance frequencies $\omega_{1,2}$ are collected in the function $R(t,T_{eff})$.

Inserting Eq. S6 in Eq. S4, the model for describing the time-evolution of the TDI beating pattern can then be written as:

$$I(\boldsymbol{q},t) \propto \left|R\left(T_{eff}\,,t\right)\right|^{2}\left[1 + \frac{2}{1+f_{\Delta E}}\,cos(\Omega\,t)\phi_{\boldsymbol{q}}(t)\right]. \qquad (S7)$$

The presence of external mechanical vibrations and defects in the absorbers may produce an inhomogeneous broadening of the spectral lines of the absorbers. This effect can be described including a fictive relaxation function which causes an additional damping of the beating pattern contrast that is independent of the sample dynamics. To this aim, a Gaussian damping function $F_D(t,\Gamma_D) = e^{\left(-t\Gamma_{Damp}\right)^2}$ acting on the beating term arising from the interference of the radiation from the upstream and downstream absorbers has been included [32-33]. An average value for the three detectors of $0.27\Gamma_0$ has been found.

In order to take in account both the finite time response of the APD detectors and the presence of a distribution of thicknesses $L$ (and thus of effective thicknesses $T_{eff}$) for both absorbers, the same procedure as in [36] has been followed.

The parameters of the reference beating pattern, that is $T_{eff}$, $\Omega$ and the fictive damping $\Gamma_{Damp}$, were fixed by fitting the TDI beating patterns collected with the sample at $T$ =25 K at $q$=13, 24 and 37 nm$^{-1}$. At such a low temperature, well below the glass-transition temperature of the sample ($T_g$=154 K), we can indeed safely assume the sample to be static in the experimental time-window of the technique.

To model the relaxation processes of the sample we used a simple stretched exponential at each temperature and exchanged wave-vector, similarly to what reported in [11, 16]:

$$\phi'_{\boldsymbol{q}}(t) = \frac{2}{1+f_{\Delta E}} \cdot f_q \, e^{-\left(\frac{t}{\tau(\boldsymbol{q},T)}\right)^{\beta_{KWW}}} = f'_q \, e^{-\left(\frac{t}{\tau(\boldsymbol{q},T)}\right)^{\beta_{KWW}}} \quad , \qquad (S8)$$

where $\tau(\boldsymbol{q},T)$ is the relaxation time, $f'_q$ is the initial beating pattern contrast, related to the relaxation strength, $f_q$, via $f_{\Delta E}$, and $\beta_{KWW}$ is the stretching parameter. It is important to stress that the experimental scheme used in this experiment did not allow us to disentangle $f_q$ and $f_{\Delta E}$, and from the fitting procedure we measured only $f'_q$ .

Since the quality of the data did not allow us to simultaneously fit the three relaxation parameters $f'_q$, $\tau$ and $\beta_{KWW}$ at all temperatures and scattering vectors, the stretching parameter $\beta_{KWW}$ was kept fixed when fitting the experimental data. In particular, the stretching parameters obtained from the analysis of the DS measurements were used to represent the shape of the relaxations at the macroscopic ($q = 0$) scale and were then scaled to represent the shape of the relaxations at higher $q$ values using the results of a numerical simulation of a model hydrogen-bonded system [40]. Specifically, the $\beta_{KWW}$ was fixed to 0.51 for all scattering vectors $q > 13$ nm$^{-1}$, that is to the value found for the $\alpha$ relaxation from the DS measurements, whereas at $q$=13 nm$^{-1}$, close to the maximum of the static structure factor $q_{max}$=14 nm$^{-1}$, $\beta_{KWW}$ was fixed to 0.64, i.e. it was increased by ≃25% with respect to the DS value.

To test the consistency of this assumption, the TDI beating patterns measured at $q$ =13 nm$^{-1}$ and at temperatures T<192 K were analyzed leaving as free parameter also the stretching parameter $\beta_{KWW}$. In fact, these data were collected in a temperature range where the entire decorrelation of the density fluctuations is observed. An average value $\beta_{KWW}$ =0.7(3) was found in this way, which is compatible with our assumption of 0.64 discussed before.

As already stated above, a stretched exponential was used in the whole investigated temperature range, including the temperatures where the $\beta_{JG}$ process was detected. This approximation, already used in other TDI measurements [11, 16], was followed in order to introduce a minimal amount of bias into the data analysis. However, it is important to notice that $\beta_{KWW} = 0.51$ is also compatible with the shape parameter which is obtained from the parameters of the Cole-Cole function used to fit the $\beta_{JG}$ peak in the DS spectra. Indeed, using the relations reported in [42] to transform the obtained Cole-Cole shape parameters into KWW ones, a value for the stretching parameter of 0.52 is found.

In Fig. S4-(a) and Fig. S5-(a) two examples of TDI beating patterns along with the fitting curves from Eq. S7 are reported. The residuals of the fitting procedures, defined as the difference between the experimental data and the model normalized to the standard deviation, are shown in Fig. S4-(b) and in Fig. S5-(b).

It is possible to notice that they are distributed according to a Gaussian curve with zero mean and unitary standard deviation, as can be inferred also from their distributions (Fig. S4-(c), Fig.S5-(c)).

Similar results were obtained for all the measured datasets, thus confirming that the used models well describe the experimental data.

## S4 Initial beating pattern contrast

More details on the temperature dependence of the initial beating time contrast, $f'_q$, can be learned looking at Fig. S6 where $1/f'_q$ is reported as a function of $T/T_g$ in the glassy state for $q$ =13, 24 and 37 nm$^{-1}$. Classically, in the harmonic approximation, it can be easily demonstrated that the inverse of the total ($\alpha + \beta_{JG}$) strength of the system linearly approaches 1 as $T \to 0$ [S5]. Since $f_{\Delta \mathrm{E}}$ approaches $f_q$ at low $T$ , this also holds for $1/f'_q$. Indeed, the difference between $f_{\Delta E}$ and $f_q$, which is due to vibrations and fast relaxations, scales at most linearly with $T$ and therefore becomes negligible at sufficiently low temperature [S6]. This has already been demonstrated to be true in glycerol [32]. The linear $T$ dependence of $1/f'(q)$ actually holds for $q$=13 nm$^{-1}$

below $T_g$, whereas it does not hold at $q$=24 nm$^{-1}$ below 70 K. At $q$=37 nm$^{-1}$ there is no temperature range below $T_g$ from where $1/f'(q)$ can be obviously extrapolated to 1.

A possible explanation of this result can be found taking into account the zero-point motions of the molecules. The temperature at which zero-point vibrations are expected to become relevant depends on the frequency of the dominant vibrational mode at a given $q$. The frequency of this dominant mode can be read out from the dispersion curve characteristic of that system, and it typically shows a sine-like oscillation on an increasing trend as a function of $q$, with a first minimum value corresponding to the end of the pseudo-Brillouin zone ($q_{max}$ =14 nm$^{-1}$ in the present case) [S7, S8]. Therefore zero-point effects are expected to be more relevant at higher temperature the larger is the $q$ value above $q_{max}$, in agreement with what here observed.


[S1] Chumakov, A. I., Sergeev, I., Celse, J.-P., Rüffer, R., Lesourd, M., Zhang, L. and Sánchez del Río, M. *J. Synchrotron Rad.* **21** ,315 (2014)

[S2] Ishikawa, T., Yoda, Y., Izumi, K., Suzuki, C. K., Zhang, X. W., Ando, M. and Kikuta S., *Rev. of Sci. Ins.* **63**, 1015 (1992)

[S3] Kremer, F., Loidl, A. Scaling of Relaxation Processes (*Springer*, Berlin), (2018).

[S4] Smirnov, G. V., Bürck, U., Franz, H., Asthalter, T., Leupold, O., Schreier E. and Petry, W. *Phys. Rev. B* **73**, 184126 (2006).

[S5] Scopigno, T., Ruocco, G., Sette, F. and Monaco, G. *Science* **302**, 849 (2003).

[S6] Sokolov, A. P. and Novikov, V. *Phil. Mag.* **84**, 1355, (2004).

[S7] Benassi, P., Nardone, M., Giugni, A., Baldi, G. and Fontana, A. *Phys. Rev. B.* **92**, 104203 (2015).

[S8] Scopigno, T., Ruocco G. and Sette, F. *Rev. Mod. Phys.*, **77**, 3 (2005).

## Supplemental figures

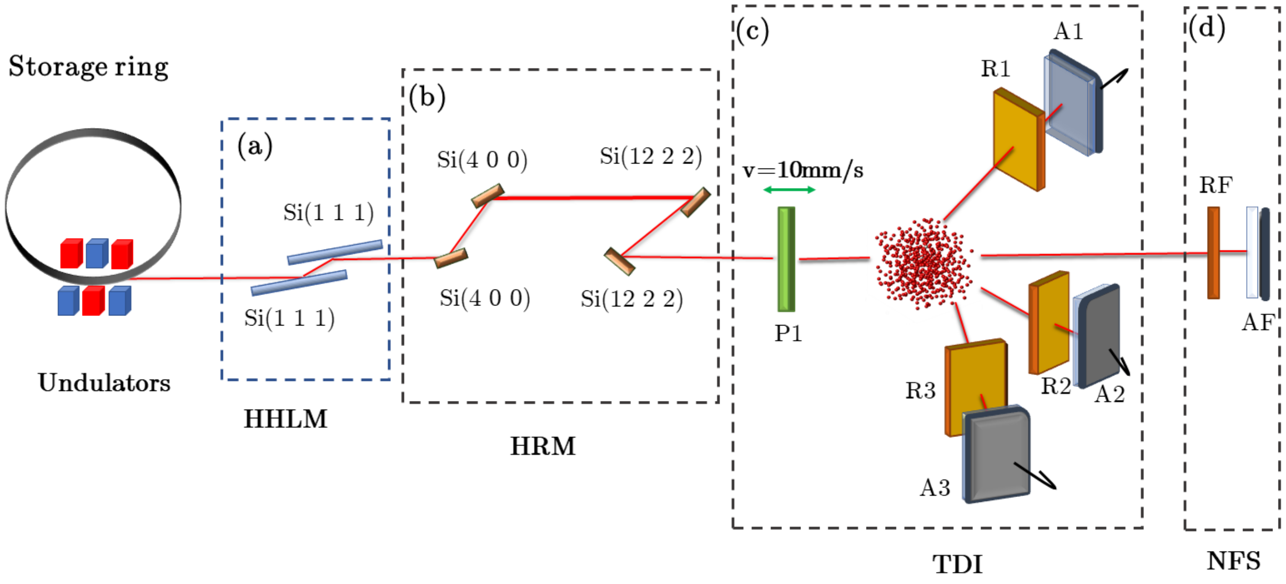


**Fig. S1. Skecth of the experimental setup used for nuclear γ-resonance time interferometry.** HHLM and HRM in (a) and (b) indicate the high heat load and high-resolution monochromator, respectively. In (c) the probe absorber is reported in green (P1), whereas the reference ones (R1, R2, R3), placed at different scattering angles together with the corresponding APD detector (A1, A2 and A3) are represented in dark yellow. PF and AF in (d) indicate the absorber and the avalanche detector used in the forward direction to monitor the stability of the experimental set-up.

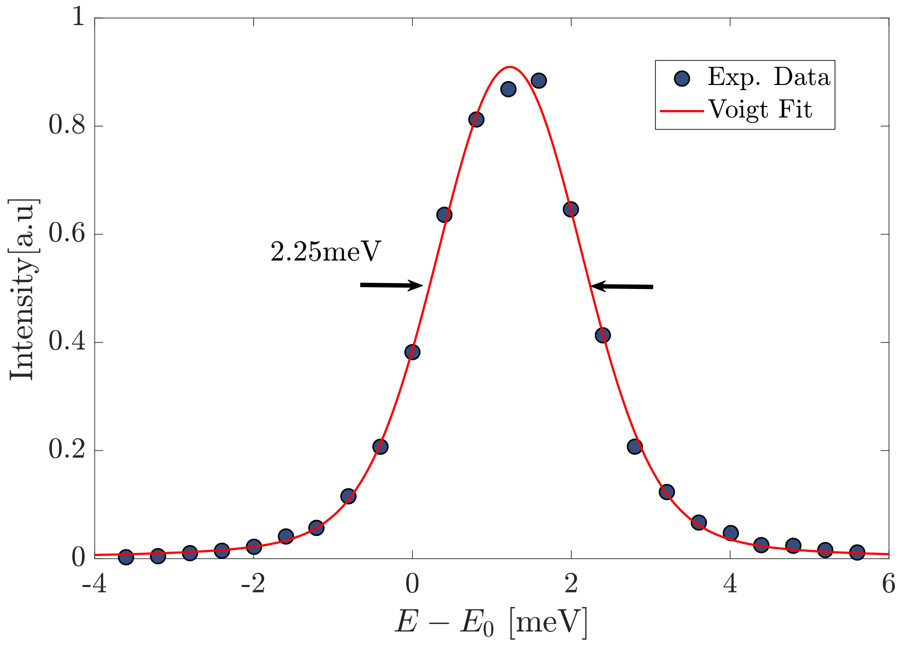


**Fig. S2**: **Energy spectrum of the incident syncrotron radiation used to excite the probe and reference nuclear absorbers of the time-domain interferometer**. Black points: experimental data. Red solid line: curve obtained from fitting a Voigt profile to the measured energy spectrum.

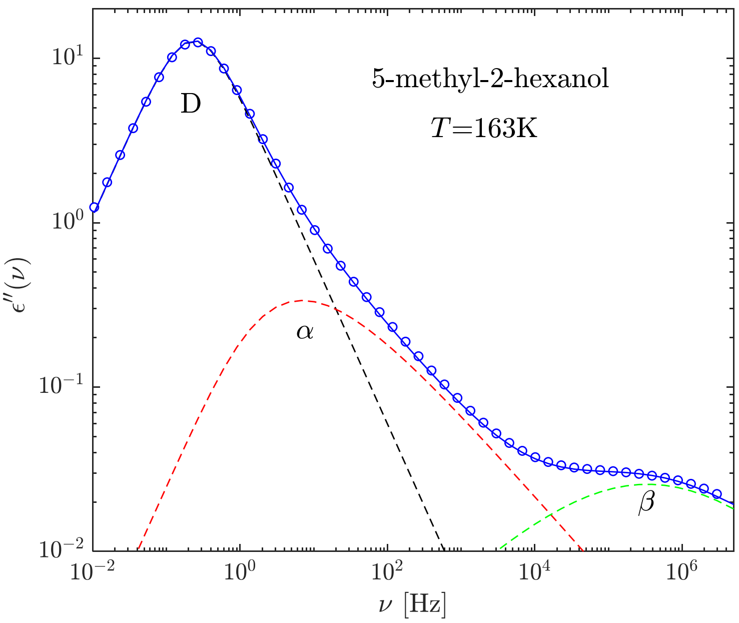


**Fig. S3: Dielectric loss spectrum of 5-methyl-2-hexanol at T=163 K.** Blue circles: experimental data; blue solid line: model curve fitted to the spectrum; black solid line: Debye peak; red solid line: structural relaxation; green solid line: Johari-Goldstein relaxation.

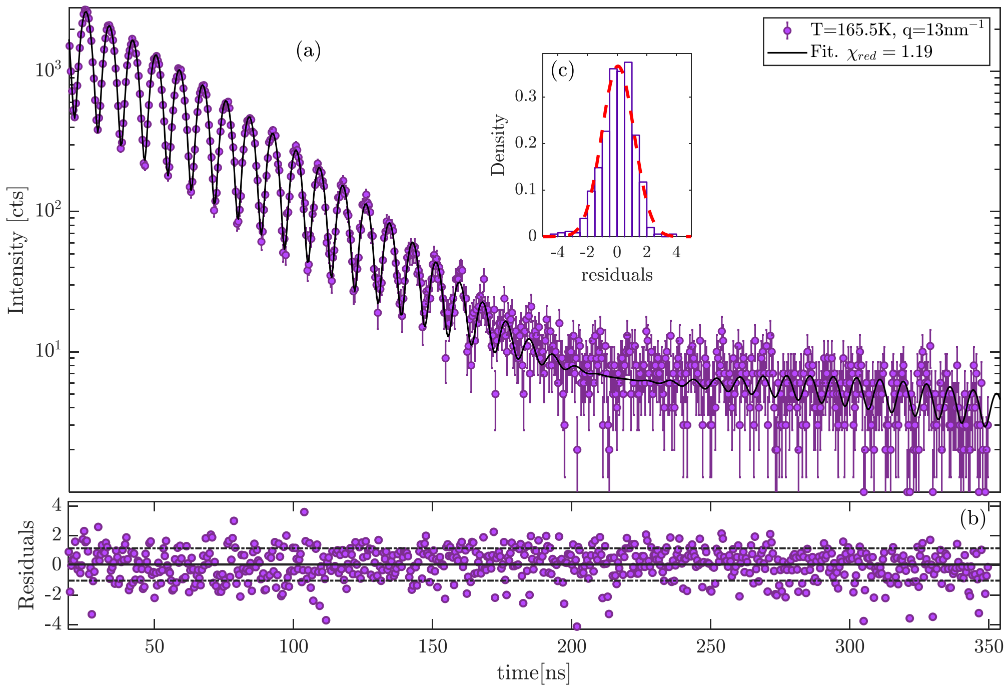


***Fig. S4.*** **Time evolution of the TDI interferogram from 5-methyl-2-hexanol at a temperature of T=165.5 K and exchanged wavevector q=13 nm$^{-1}$**. (a): liliac circles with errorbars: experimental data; black solid line: model fitted curve to the time pattern; (b) normalized residuals of the fitting procedure. Liliac circles: normalized residuals. Black solid line: mean of the residual distribution. Black dashed lines: standard deviation of the distribution of the residuals. (c) Calculated distribution of the residuals. Liliac bars: histogram of the residuals normalized in area. Red solid line: curve obtained from fitting a Gaussian to the histogram of the residuals.

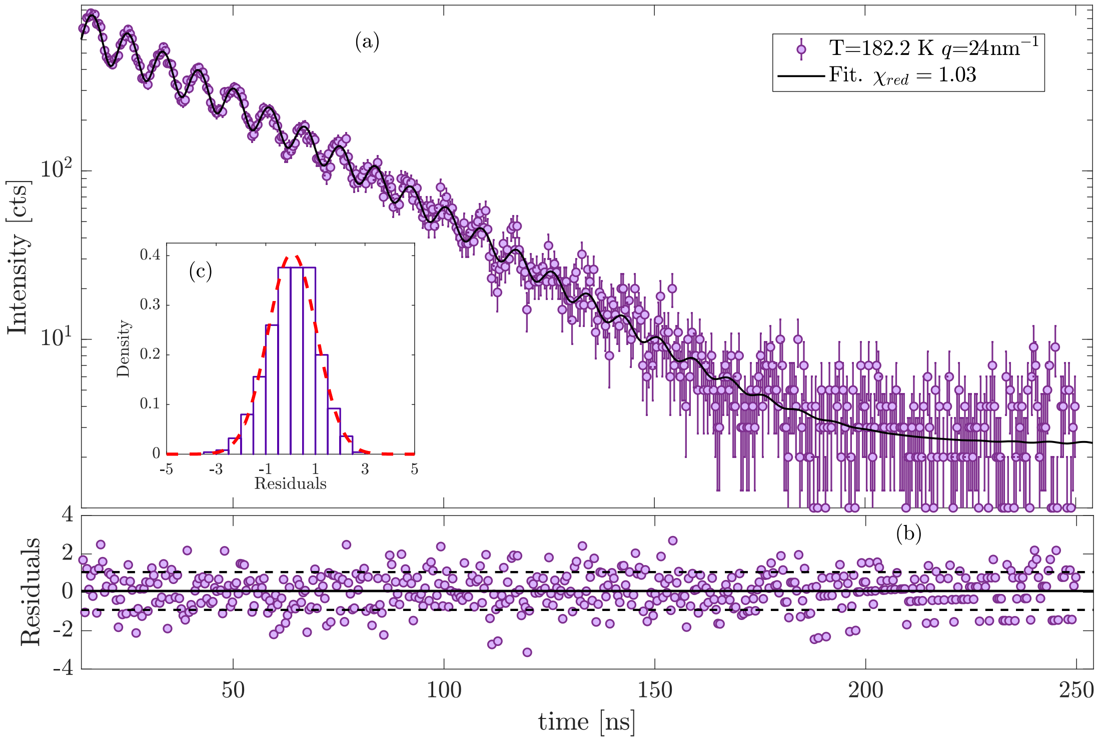


**Fig. S5. Time evolution of the TDI interferogram from 5-methyl-2-hexanol at a temperature of T =182.2 K and exchanged wavevector of q=24 nm$^{-1}$.** Same plots and symbols as in Fig. S4.

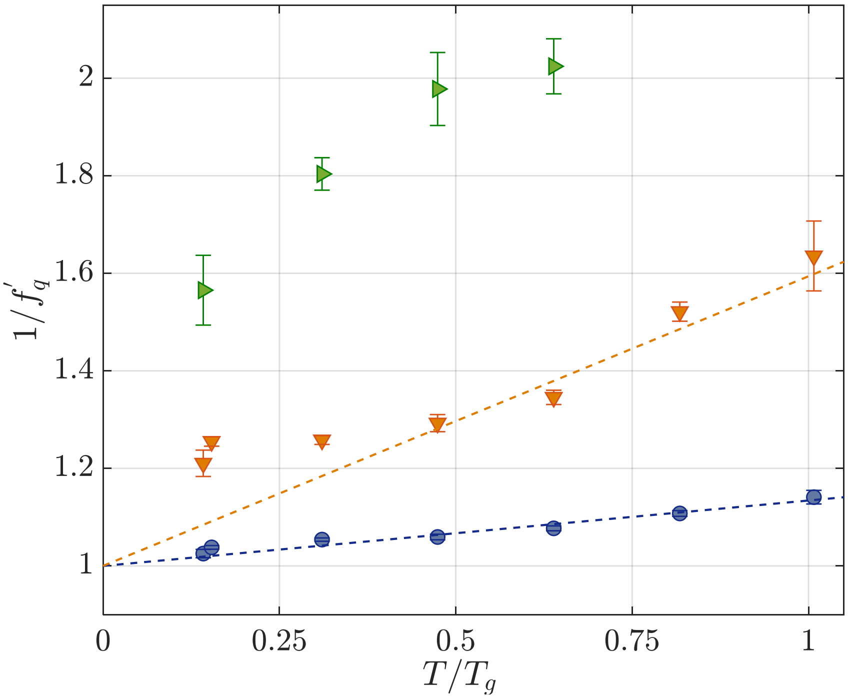


**Fig. S6. Low temperature dependence of the initial contrast.** The inverse of the initial beating time contrast, $f_q'$ is reported as a function of $T/T_g$. The dashed lines are linear fits with intercept at 1. Blue circles, orange downward pointing triangles, green left pointing triangles correspond to the values obtained respectively for $q$=13, 24, 37 nm$^{-1}$.
